# Electrochemical Reduction of CO_2_ on Hollow Cubic Cu_2_O@Au Nanocomposites

**DOI:** 10.1186/s11671-019-2892-3

**Published:** 2019-02-21

**Authors:** Wei Tan, Bo Cao, Wenqiu Xiao, Min Zhang, Shoushan Wang, Shilei Xie, Dong Xie, Faliang Cheng, Qingquan Guo, Peng Liu

**Affiliations:** 1 Guangdong Engineering and Technology Research Center for Advanced Nanomaterials, School of Environment and Civil Engineering, Dongguan University of Technology, Dongguan, 523808 China; 2 School of Chemical Engineering and Light Industry, Guangdong University of Technology, Guangzhou, 510006 China

**Keywords:** CO_2_ electrochemical reduction, Cuprous oxide, Hollow cubes, Metal-oxide interface

## Abstract

Surfactant-free and low Au loading Cu_2_O@Au and Au hollow cubes, based on electrodeposited Cu_2_O cubes as sacrificed templates, were prepared by means of a galvanic replacement reaction (GRR). The electrocatalytical performance of the as-prepared catalysts towards carbon dioxide (CO_2_) electrochemical reduction was evaluated. The experimental results show that Cu_2_O@Au catalyst can convert CO_2_ to carbon monoxide (CO) with a maximum Faradaic efficiency (FE) of ~ 30.1% at the potential of − 1.0 V (vs. RHE) and is about twice the FE of the other catalysts at the same potential. By comparison, such electrocatalytical enhancement is attributed to the metal-oxide interface in Cu_2_O@Au.

## Background

CO_2_ is considered as the main greenhouse gas that contributes to global warming; therefore, finding an effective way to convert/store CO_2_ has attracted more and more attention [1, 2]. The main methods for reducing CO_2_ concentration in the atmosphere include CO_2_ capturing and storing it underground [3, 4] or convert it to added value chemicals [5–7]. Due to the stable chemical properties of CO_2_, it is necessary to use high temperature, high pressure, or catalyst to make it reactive. Considering the energy and economy cost, electrochemical conversion of CO_2_ under mild conditions is a promising strategy for decreasing the excess greenhouse gas and achieving an artificial carbon cycle [8–10]. However, the major difficulties in CO_2_ electrochemical reduction are the intrinsic stability of CO_2_, the lower potential of CO_2_ reduction reaction (CO_2_RR), and the low selectivity for reduction products [11]. Thus, it is urgent to develop CO_2_ reduction catalysts with high selectivity, good stability, and excellent activity.

Previous studies show several metallic electrodes, such as Au, Ag, Cu, Pd, and Sn, are attractive candidates for CO_2_RR [12]. Among them, copper is the only metal catalyst which is found to produce considerable C1–C3 hydrocarbon products and alcohols [13]. Au, which is a highly active catalyst towards CO_2_ electrochemical reduction, can produce CO from CO_2_ with high selectivity and low overpotential [11]. Except for Cu and Au, the other metal electrodes including Ag, Pd, and Sn primarily convert CO_2_ to CO or formate (HCOO^−^) via a two-electron transfer pathway [14–17]. However, on the one hand, it is difficult to improve the selectivity and stability of Cu-based catalysts towards the electrochemical reduction of CO_2_ to C1–C3 hydrocarbon products. On the other hand, Au is highly selective for CO production, but its high cost and rare-earth abundance hinder its industrialization in CO_2_RR [18, 19]. The composites based on copper and gold are of great potential for CO_2_ electrochemical reduction. But most of the currently reported CuAu catalysts were synthesized via solvothermal method [20]. The morphology of the nanoparticles is difficult to control, and these particles tend to be easily oxidized and aggregated [21, 22]. Therefore, it is very important to develop a kind of gold and copper composite with controllable morphology, high stability, and high product selectivity for CO_2_ electrochemical reduction. Besides, it is reported that the metal-oxide interface could improve the electrocatalytical activity of the catalysts towards CO_2_RR [23].

Here in this paper, we report a surfactant-free Cu_2_O@Au nanocomposite in which Cu_2_O/Au interface was constructed for electrocatalytical reduction of CO_2_ in water. For comparison, the hollow cubic Au catalysts were prepared by dissolving Cu_2_O in Cu_2_O@Au catalysts in ammonia. The experimental results showed that the metal/oxide interface in Cu_2_O@Au catalyst could activate inert CO_2_ molecule and increase the FE of CO. The CO FE is 30.1% on Cu_2_O@Au electrode at − 1.0 V (vs. RHE) which is twice than that on Cu_2_O and Au electrodes prepared in this work. This result not only proved the metal-oxide interface could improve the electrocatalytical activity of the electrodes towards CO_2_RR, but also paved the way for metal-oxide catalyst synthesis.

## Methods

### Materials

Copper (II) trifluoroacetate (Cu (TFA)_2_, 98%), potassium trifluoroacetate (KTFA, 98%), and chloroauric acid (HAuCl_4_, 99.9%) were purchased from Sigma-Aldrich and used directly without any purification. All solutions were prepared with Milli-Q ultrapure water (Millipore ≥ 18.2 MΩ cm). Nitrogen (N_2_) (99.999%) and CO_2_ (99.999%) gases used in the experiment were purchased from Foshan MS Messer Gas CO., Ltd. Carbon paper which thickness is 0.3 mm was purchased from Hesen in Shanghai.

### Preparation of Cu_2_O Nanocubes and Cu_2_O@Au

Cu_2_O nanocubes were synthetized according to the method reported in previous literature [24]. The cubic Cu_2_O nanoparticles were electrodeposited on a carbon paper (1 cm × 1 cm) using chronoamperometry at − 0.06 V (vs. SCE) for 1 h in 10 mM Cu (TFA)_2_ and 0.2 M KTFA solution. Before the Cu_2_O nanocubes electrodeposition, the carbon paper was washed by water and ethanol several times.

The preparation of Cu_2_O@Au composite was immersing Cu_2_O cubes into 2 mL HAuCl_4_ (1 mM) solution for 30 min at 277 K.

### Preparation of Hollow Cubic Au

The as-prepared Cu_2_O@Au composite was immersed in 2 M ammonia water for 12 h at 277 K to remove Cu_2_O and retain hollow cubic Au on the carbon paper.

### Characterization

The morphologies and structures of nanomaterials were characterized by scanning electron microscopy (SEM, JEOL-6701F) equipped with an energy dispersive X-ray (EDX) detector system. The X-ray diffraction (XRD) patterns were recorded using Rigaku Ultima IV X-ray diffractometer with Cu Kα radiation (λ = 1.5406 Å) to study the compositions of the products.

### Electrochemical Measurements of CO_2_

**Fig. 1:**
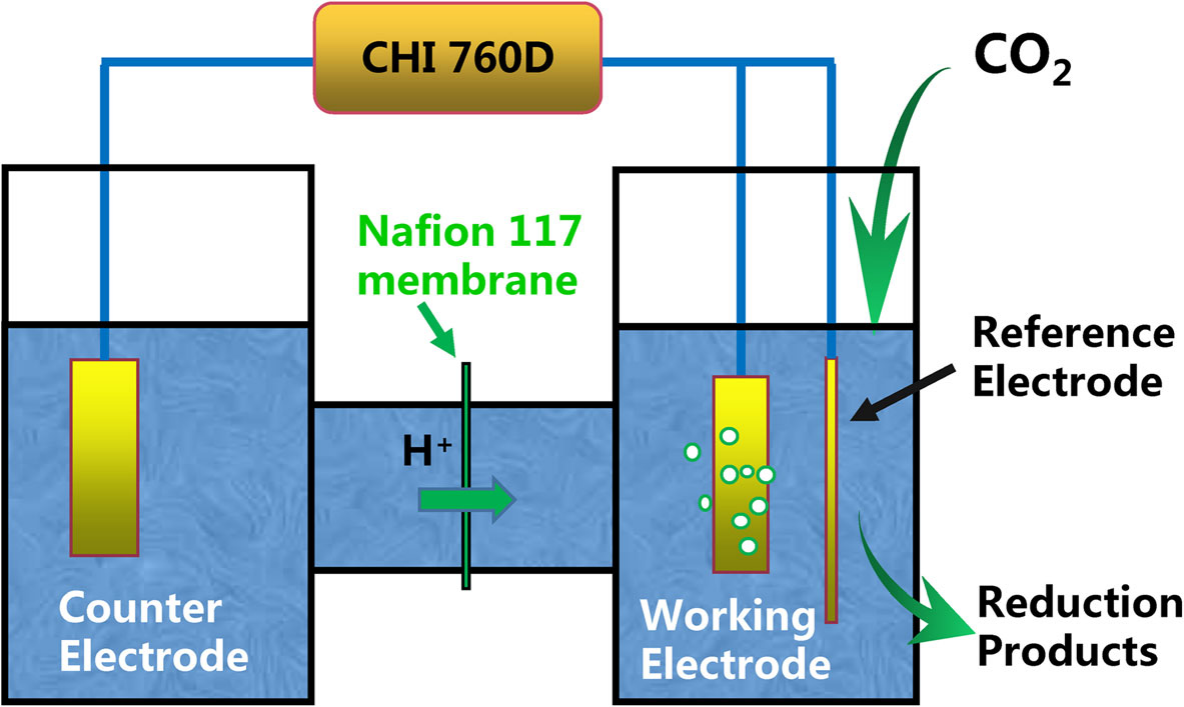
A diagrammatic sketch of the H-type electrochemical cell

Electrochemical measurements were carried out with a CH Instruments 760D (Chenhua, Shanghai) and a three-electrode system. The CO_2_ electrochemical reduction was carried out in a two-compartment H-type cell with an Ag/AgCl and a platinum sheet (1 cm × 1 cm) used as reference and counter electrodes, respectively. Compensation for 85% iR drop was used in CO_2_ electrochemical reduction. In this work, all potentials reported in the CO_2_ electrochemical reduction were referenced against the reversible hydrogen electrode (RHE). The RHE used the following conversion: *E*_RHE_ (V) = *E*_Ag/AgCl_ (V) + 0.197 V + (0.059 V × pH) [25]. A diagrammatic sketch of the H-type electrochemical cell is shown in Fig. 1. The two electrochemical cells were separated by a proton exchange membrane (Nafion 117, Sigma-Aldrich).

Linear sweep voltammetry (LSV) experiments were performed in 0.1 M KHCO_3_ solution under N_2_ (99.999%) or CO_2_ (99.999%) atmosphere. Prior to LSV tests, N_2_ or CO_2_ were purged into the solution in H-type electrochemical cell for 20 min, respectively.

Before CO_2_RR experiments, the electrolyte solution was saturated for 20 min with CO_2_ and the pH of the 0.1 M KHCO_3_ solution was about 8.6. The CO_2_ electrochemical reduction was performed under potentiostatic conditions while the current and product concentration were monitored. The as-prepared materials were used as working electrodes. The CO_2_RR experiments were repeated thrice at each potential. Detection of CO_2_ reduction products were used by an online gas chromatography (GC Agilent, 7890B). A GC run was conducted every 930 s. The GC was equipped with two Plot-Q columns, a thermal conductivity detector (TCD), a flame ionization detector (FID), and a demethanizer with N_2_ (99.999%) as carrier gas. The contents of liquid products were neglected in this work. During the CO_2_RR experiments, CO_2_ was vented into the cathode electrolysis cell at a flow rate of 20 ml min^−1^ continuously.

1 $$ {i}_x=\frac{C_x\cdot q\cdot p}{RT}\cdot {n}_xF $$

2 $$ \mathrm{FE}\left(\%\right)=\frac{i_x}{i_{total}}\cdot 100 $$

The FE calculation equation is shown in Eqs. 1 and 2, in which *i*_total_ is the current density recorded by the potentiostat during CO_2_RR [26]. The partial current (*i*_*x*_) which is needed to generate each product (*x* = H_2_, CO, CH_4_, C_2_H_4_) is derived from Eq. 1. *C*_*x*_ is extracted from the calibration curve GC volume concentration of product *x*. *n*_*x*_ is the number of reduced electrons required to produce *x* from carbon dioxide molecules. *q* is the gas flow rate, *p* is constant pressure, and *T* is the room temperature. *R* is the gas constant, and *F* is the Faradaic constant.

## Results and Discussion

### Morphology

**Fig. 2:**
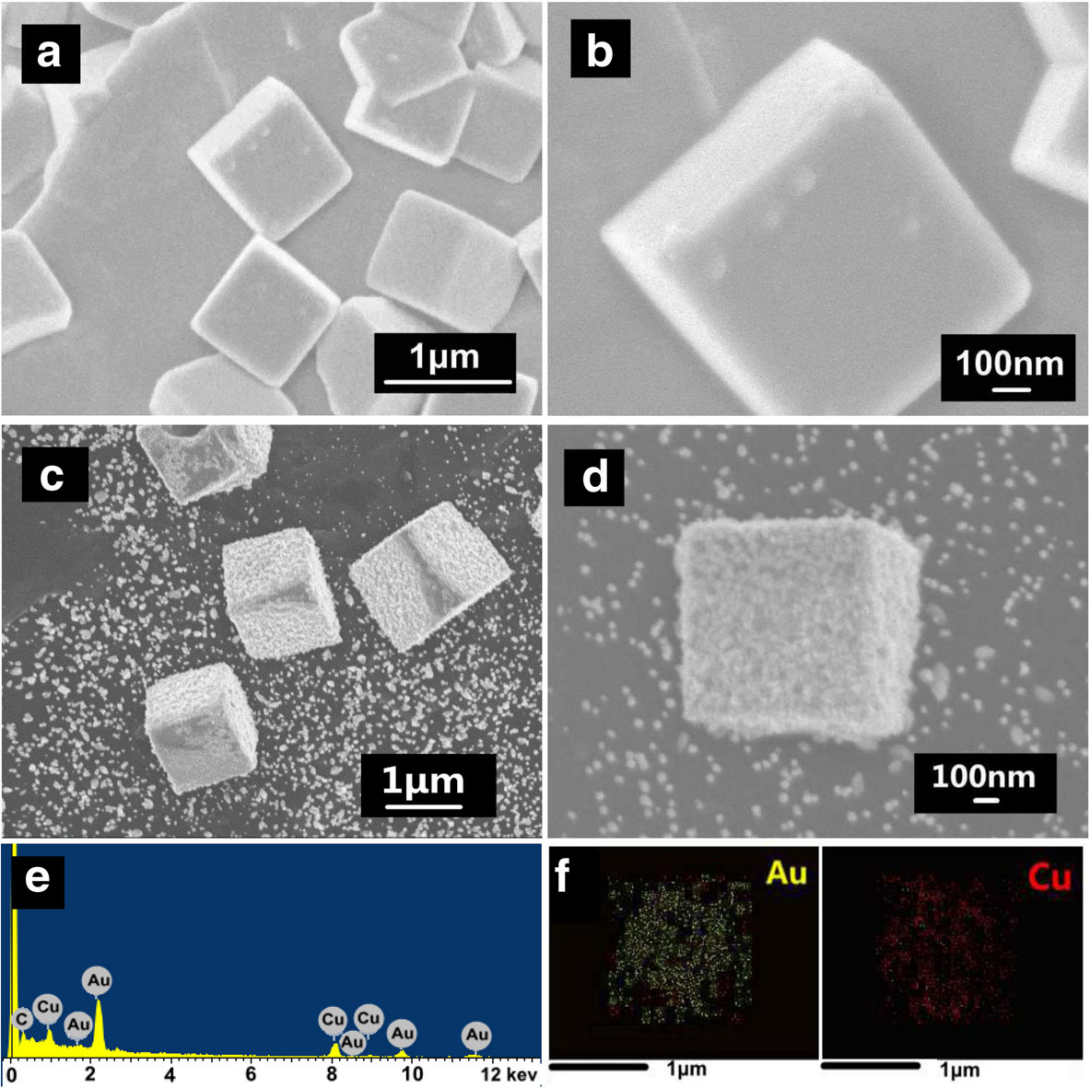
The SEM images of Cu_2_O nanocubes (**a**, **b**), Cu_2_O@Au nanoparticles (**c**, **d**), and EDX of Cu_2_O@Au nanoparticles (**e**, **f**)

The morphologies and structures of as-prepared Cu_2_O and Cu_2_O@Au nanocubes characterized by SEM were shown in Fig. 2. The Cu_2_O nanocubes electrodeposited on the carbon paper had regular shapes and smooth surface (Fig. 2a). The average edge length of the Cu_2_O cubes was about 1 μm as shown in Fig. 2b. An appropriate reaction time and Au^3+^ solution concentration of GRR on Cu_2_O nanoparticles would produce Cu_2_O@Au nanostructures as shown in Fig. 2c and d.

After Cu_2_O nanocubes were immersed in HAuCl_4_ (1 mM) solution for 30 min, the surface distribution of Au and Cu of Cu_2_O@Au composites was examined by EDX mapping shown in Fig. 2e and f. It showed that Au nanoparticles were uniformly distributed on the Cu_2_O nanocube surface. The GRR between Cu_2_O and HAuCl_4_ involves the evolution of an internal hollow core and surface precipitation of Au nanoparticles [27, 28].

**Fig. 3:**
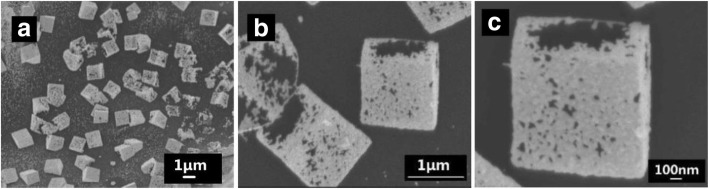
The SEM images of hollow cubic Au (**a**–**c**) of different magnification

As shown in Fig. 3, Cu_2_O in Cu_2_O@Au composites was removed and the retained Au nanoparticles inherit the cubic frame of the Cu_2_O@Au composites, after Cu_2_O@Au nanocubes were immersed in ammonia water for 12 h. The small Au nanoparticles in hollow cubic Au framework were about 20~30 nm in diameter.

### XRD Analysis

**Fig. 4:**
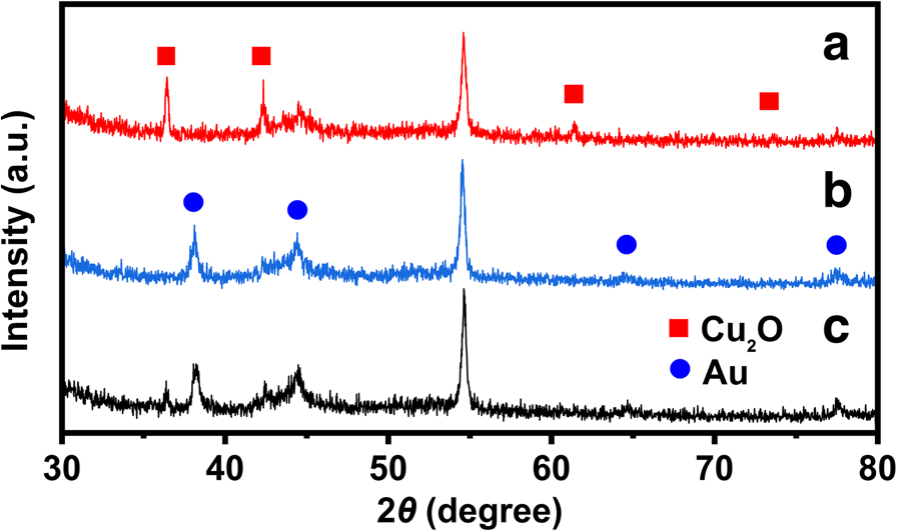
XRD patterns of (a) Cu_2_O cube, (b) hollow cubic Au, and (c) Cu_2_O@Au

The crystal structure of the as-prepared catalysts was investigated by XRD, and the diffraction patterns were shown in Fig. 4. The diffraction peak at 2*θ =* 54.51° belongs to the carbon paper. The diffraction peaks at 2*θ* = 36.46, 42.36, 61.44, and 73.55° are ascribed to the (111), (200), (220), and (311), respectively, of the Cu_2_O cube (JCPDS 78-2076). Four weak peaks at 2*θ =* 38.18, 44.39, 64.57, and 77.54° are assigned to the (111), (200), (220) and (311), respectively, of Au (JCPDS 04-0784) which replaced Cu_2_O on the carbon paper. Most of Cu_2_O were substituted by Au; thus, the diffraction peaks corresponded to Cu_2_O disappeared in XRD pattern of hollow cubic Au.

### CO_2_ Electrochemical Reduction Performance

**Fig. 5:**
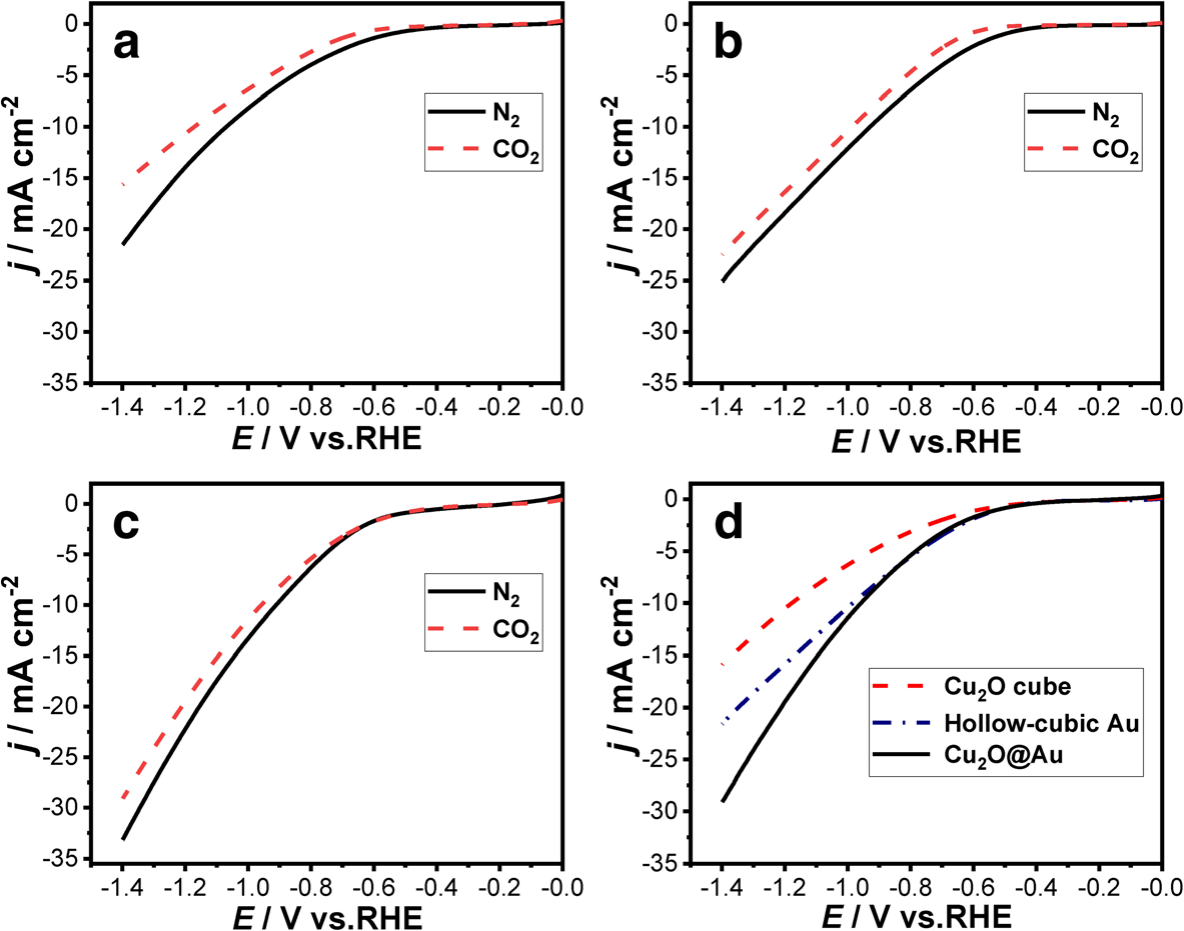
LSV curves obtained on **a** Cu_2_O cube, **b** hollow cubic Au, and **c** Cu_2_O@Au electrodes in N_2_-saturated (black solid line) and CO_2_-saturated (red dotted line) 0.1 M KHCO_3_ solutions. **d** LSV curves of the three samples in CO_2_-purged 0.1 M KHCO_3_ solutions

The LSV curves of Cu_2_O cube, Cu_2_O@Au, and hollow cubic Au electrodes are shown in Fig. 5. The LSV experimental condition was obtained at a cathodic sweeping rate of 50 mV s^−1^ with N_2_-saturated or CO_2_-saturated 0.1 M KHCO_3_ solution. The current density of all the three samples under the N_2_ atmosphere is higher than that under CO_2_; this difference may be caused by the hydrogen evolution reaction (HER) on Cu_2_O cube, Cu_2_O@Au, and hollow cubic Au, i.e., with continuous flow of CO_2_ in the cathodic electrolytic cell, the surface of the electrode is covered by adsorbed CO molecules which will inhibit the HER on electrode surface and decrease the reduction current [29]. The current density of Cu_2_O@Au electrode in CO_2_-saturated 0.1 M KHCO_3_ solution is higher than Cu_2_O and hollow cubic Au electrodes as shown in Fig. 5d.

**Fig. 6:**
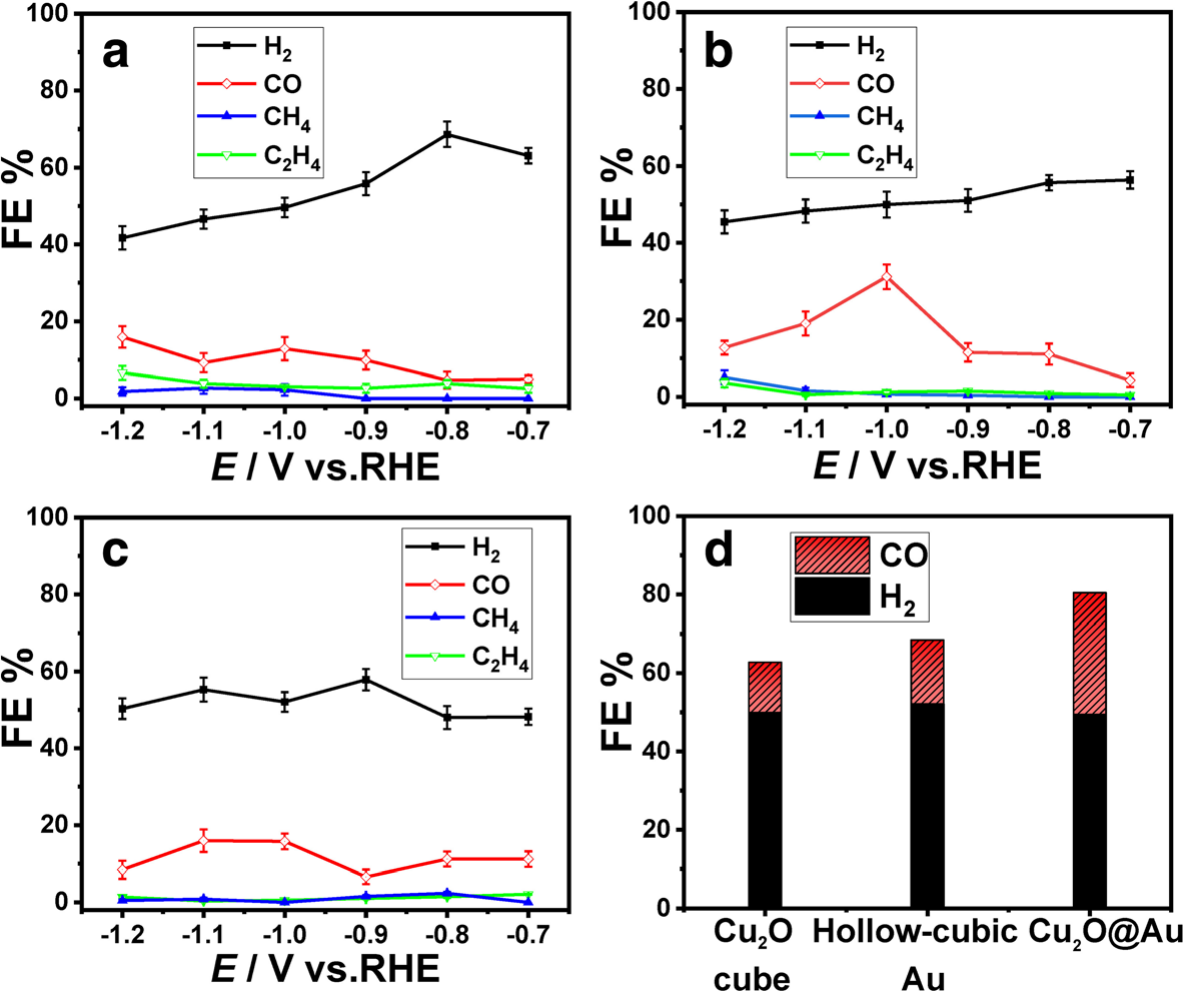
FE of **a** Cu_2_O cube catalyst, **b** Cu_2_O@Au catalyst, and **c** hollow cubic Au catalyst. **d** Comparison of FE for CO and H_2_ at − 1.0 V vs RHE on three catalysts

The electrochemistry method of amperometric *i*–*t* was used to evaluate the performance of CO_2_RR in 0.1 M KHCO_3_ solution at room temperature under atmospheric pressure. The potentials are set between − 0.7 and − 1.2 V for subsequent product determination. At different potentials, the FE of H_2_ and CO for CO_2_RR on Cu_2_O cubes have a significant difference, as shown in Fig. 6a, i.e., the FE of H_2_ is decreasing because the surface of Cu_2_O cubes is covered by CO molecules produced by CO_2_RR, and the HER is inhibited [30]. The FE of CH_4_ and C_2_H_4_ vary slightly in different potentials.

The FE of Cu_2_O@Au catalyst is shown in Fig. 6b. The FE of CO keeps upward trend with potential decreasing and reaches a maximum of 30.1%, at − 1.0 V (vs. RHE). The FE of H_2_ decreases from 56.7 to 45.6%. Compared with the Cu_2_O@Au catalyst, the maximum CO FE of hollow cubic Au catalyst is 16.3% at − 1.0 V (Fig. 6c). The CO FE of Cu_2_O@Au catalyst at − 1.0 V is about twice of hollow cubic Au catalyst at the same potential. Cu_2_O@Au composite shows superior catalytic activity for CO_2_ electrochemical reduction than Cu_2_O cube catalyst and hollow cubic Au catalyst, and it is related to the interfacial effect of metal oxides.

To understand the reaction mechanism on CO_2_RR to CO, we considered the following reaction steps:

3 $$ {\mathrm{CO}}_2\left(\mathrm{g}\right)+\ast +{\mathrm{H}}^{+}\left(\mathrm{aq}\right)+{\mathrm{e}}^{-}{\to}^{\ast}\mathrm{COOH} $$

4 $$ {}^{\ast}\mathrm{CO}\mathrm{OH}+{\mathrm{H}}^{+}\left(\mathrm{aq}\right)+{\mathrm{e}}^{-}{\to}^{\ast}\mathrm{CO}+{\mathrm{H}}_2\mathrm{O}\left(\mathrm{l}\right) $$

5 $$ {}^{\ast}\mathrm{CO}\to \mathrm{CO}\left(\mathrm{g}\right)+\ast $$

Generally, Eq. 3 is perceived as the potential limiting step on CO_2_RR to CO [23]. The corresponding binding energy can be substantially lowered on the interface of Cu_2_O@Au, compared to the Cu_2_O cube surface or Au surface. In addition, the Eq. 4 and Eq. 5 are also facilitated at the Cu_2_O@Au interface. It indicates that the interfacial effect of metal oxides could enhance the CO_2_ adsorption and the electrochemical surface area [31, 32]. The Cu_2_O@Au catalyst consists of Cu_2_O and Au nanoparticles can supply a metal-oxide interface to activate inert CO_2_ molecules, enhance charge transfer efficiency, and increase FE of CO [33].

Compared to the mass transfer effect of hollow cubic Au catalyst composed by Au nanoparticles, the synergistic interactions of metal oxides fabricated by Cu_2_O cubes and Au nanoparticles are more advantageous to convert CO_2_ into CO by CO_2_ electrochemical reduction.

The FE comparison for CO and H_2_ at − 1.0 V vs RHE on Cu_2_O cube catalyst, Cu_2_O@Au catalyst, and hollow cubic Au catalyst is shown in Fig. 6d. The H_2_/CO ratio of these three catalysts is as follows: 3.9, 3.2, and 1.7. The Cu_2_O@Au catalyst production ratio of 1.7 by CO_2_ electrochemical reduction is closest to that of syngas (the mixture of CO and H_2_) ratio of 2 [34, 35]. The catalyst surface construct method and the proportion of product gases would contribute to design highly selective CO_2_RR catalysts.

**Fig. 7:**
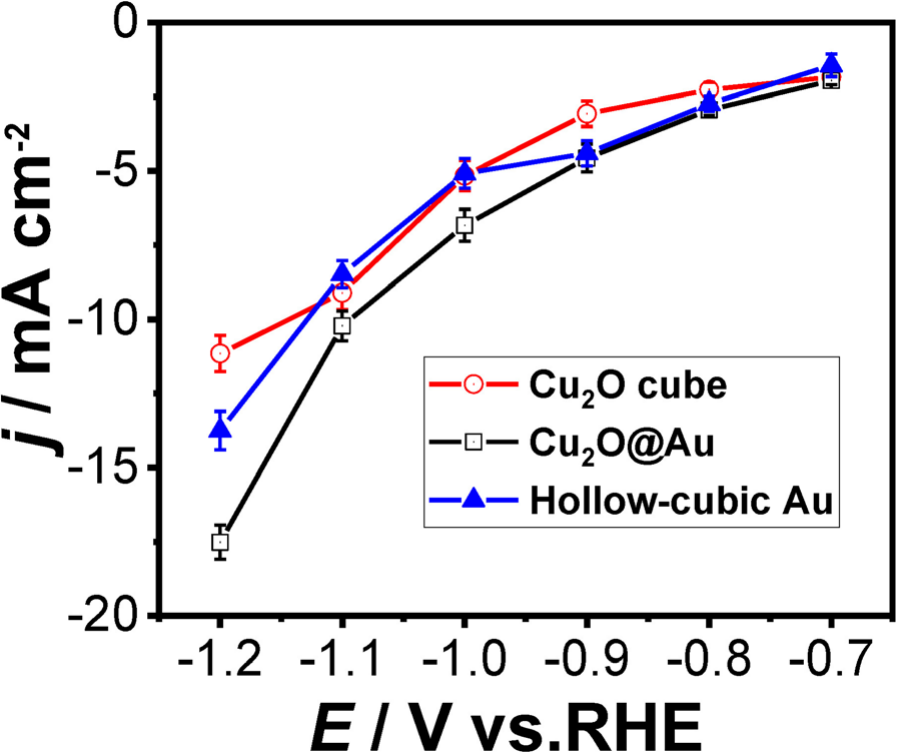
The average total current density of the three catalysts for CO_2_ reduction at different potentials

The average current density of three catalysts, which were performed by amperometric *i*–*t*, is shown in Fig. 7. With the potential increase, it shows evidently increasing current densities of three catalysts as expected. The difference of the average total current density between hollow cubic Au (blue solid line) and Cu_2_O@Au (black solid line) expands at − 1.0 V. However, the difference of the average total current density between hollow cubic Au and Cu_2_O cube (red solid line) is not marked within − 0.7 to − 1.1 V. Consequently, we could conclude that the charge transfer efficiency of Cu_2_O@Au catalyst is higher than the other two catalysts.

## Conclusions

In summary, surfactant-free and low Au loading electrodes for CO_2_ electrochemical reduction were prepared by electrodeposition and GRR. The Cu_2_O@Au catalyst shows superior catalytic activity for CO_2_RR than Cu_2_O cubes and hollow cubic Au catalyst due to the metal-oxide interface, i.e., the metal-oxide interface could activate the inert CO_2_ molecules absorbed on electrodes. For Cu_2_O@Au catalyst, it can convert CO_2_ to CO with a maximum FE of ~ 30.1% at − 1.0 V and is about twice of the other catalysts at the same potential. The produced gas of Cu_2_O@Au catalyst by CO_2_ electrochemical reduction has a H_2_/CO ratio of 1.7, which is close to the syngas ratio of Fischer–Tropsch process of 2. Based on these results, we can draw some conclusions that the Cu_2_O@Au catalyst fabricated by Cu_2_O cubes and Au nanoparticles could form a metal/oxide interface to activate inert CO_2_ molecules and this catalyst could be applied to syngas production by CO_2_ electrochemical reduction.

## Abbreviations

CO: Carbon monoxide
CO_2_: Carbon dioxide
CO_2_RR: CO_2_ reduction reaction
EDX: Energy dispersive X-ray
GC: Gas chromatography
GRR: Galvanic replacement reaction
HCOO^−^: Formate
HER: Hydrogen evolution reaction
LSV: Linear sweep voltammetry
N_2_: Nitrogen
RHE: Reversible hydrogen electrode
SEM: Scanning electron microscopy
XRD: X-ray diffraction
